# Vitamin D and bone metabolism in Graves’ disease: a prospective study

**DOI:** 10.1007/s40618-022-01927-y

**Published:** 2022-09-27

**Authors:** S. Khamisi, M. Lundqvist, A. J. Rasmusson, B. E. Engström, F. A. Karlsson, Ö. Ljunggren

**Affiliations:** 1 Department of Endocrinology and Diabetes, Uppsala University Hospital, 751 85 Uppsala, Sweden; 2 Department of Medical Sciences, Uppsala University, Uppsala, Sweden

**Keywords:** Thyrotoxicosis, 25-Hydroxyvitamin D3 (25OHD3), 1,25-Dihydroxyvitamin D3 (1,25OH2D3), Vitamin D binding protein, Osteoporosis

## Abstract

**Purpose:**

Vitamin D and osteoporosis in Graves’ disease (GD) have been examined in cross-sectional studies with divergent results. Here, we prospectively studied vitamin D metabolism and bone health in patients with newly diagnosed GD.

**Methods:**

Thirty consecutive patients with de novo overt thyrotoxicosis diagnosed with GD were included. At diagnosis, none of the patients were treated with vitamin D or anti-osteoporotic drugs. All patients were initially treated with antithyroid drugs. Blood samplings were taken at baseline and at 6 weeks, 3, 6, 12 and 24 months after treatment start. Serum levels of 25OHD3, 1,25OH2D3, calcium, parathyroid hormone (PTH), and C-terminal telopeptides of Type I collagen (CTX-I) were analysed. Bone mineral density (BMD) was measured at baseline, and 1 and 2 years after treatment initiation.

**Results:**

At diagnosis, patients with GD did not have vitamin D deficiency. There were no significant correlations between levels of 25OHD3 and thyrotoxicosis. Upon treatment of the thyrotoxicosis, serum calcium fell transiently, and PTH and 1,25OH2D3 increased. 25OHD3 fell within the normal range and stabilised at 6 months. CTX-I fell over 12 months, BMD increased significantly up to 2 years, *p* = 0.002, < 0.001 and 0.005 in the spine, left total hip and left femoral neck, respectively.

**Conclusions:**

The present data underline that thyrotoxicosis has a negative impact on bone health and demonstrate fine-tuned dynamics in bone and vitamin D metabolism. Upon treatment, bone health improved over a follow-up period of 24 months despite rising PTH. Increased conversion of 25OHD3 to 1,25OH2D3 occurs during treatment of GD.

## Introduction

Graves’ disease (GD) is an autoimmune form of hyperthyroidism with an annual incidence of 21 in 100, 000 individuals [1]. There is a genetic background that makes the disease associated with other autoimmune diseases and is more common in women, but the cause of the disease is unknown [2–4]. Hyperthyroidism often results in secondary osteoporosis by acceleration of bone resorption, causing loss of bone mineral density [5–9]. Even subclinical hyperthyroidism is a risk factor for osteoporosis [10]. It is generally believed that T3 stimulates osteoclastic activity and that an elevated thyroid hormone level is the primary cause of bone loss [11]. However, bone remodelling is influenced by many factors, such as calcium homeostasis, parathyroid hormone (PTH), vitamin D status and sex hormones. All of these are potential factors that might be influenced by thyrotoxicosis, making the exact mechanism causing bone loss in GD unknown.

Epidemiological studies have reported associations between vitamin D (25OHD3) deficiency and the risk of developing a variety of diseases, such as cardiovascular diseases, cancer, diabetes and infectious diseases [12–19]. In addition, in autoimmune diseases, such as multiple sclerosis, vitamin D deficiency has been reported as a risk factor [20–23]. With GD, the literature is not conclusive regarding vitamin D deficiency as a risk factor. In a study with thirty hyperthyroid patients in India, 26% of the study subjects had vitamin D deficiency, i.e., levels of 25OHD3 less than 25 nmol/L [24]. Yamashita et al*.* have also reported vitamin D deficiency in 40% of women and in 18% of men having GD from Japan [25]. In another study from Japan, Doi et al*.* showed that 35% of patients with thyrotoxicosis had vitamin D deficiency [26]. Other studies have also shown a significant impact of 25OHD3 deficiency on bone structure and bone metabolism [27–29]. However, in a Swedish study, patients with GD had lower 25OHD3 levels compared to the general population (55.0 ± 23.2 vs 87.2 ± 27.6 nmol/L). However, vitamin D levels were not associated with laboratory or clinical parameters of GD [30]. All studies of vitamin D and GD mentioned above were cross-sectional and focused only on 25OHD3. Levels of the active metabolite, 1,25OH2D3, in GD have not been described.

In the present study, we have prospectively monitored vitamin D metabolism and calcium homeostasis in patients with newly diagnosed GD, taking into consideration the severity of thyrotoxicosis and changes in bone mineral density.

## Materials and methods

### Study subjects

Patients with de novo GD, diagnosed by decreased levels of thyroid stimulating hormone (TSH) and positive autoimmune antibodies against TSH-receptors (TRAb), were recruited at the Uppsala University Hospital from February through November 2017. Inclusion criteria: males and females, aged 30–80 years, with newly diagnosed GD. Exclusion criteria**:** pregnant or planning to be pregnant during the study, secondary osteoporosis, corticosteroids treatment in the last 2 years as well as vitamin D and/or calcium supplementation. TRAb was below the reference range (1.7 IE/L, reference < 1.75) in one patient. This patient otherwise had laboratory findings and symptoms typical of GD, with a high and homogeneous uptake at scintigraphy and was included in the study. Two patients started with vitamin D supplementation without prescription after the baseline visit. One patient had osteoporosis at time of diagnosis and started treatment with bisphosphonate and calcium with vitamin D. These three patients were excluded from the follow-up analyses.

The study comprised six visits. During the first visit (baseline), all patients underwent an examination, including recording of demographic characteristics (sex and age), medical history, family history of fracture, concomitant medication, weight and height, and a physical examination. Blood samplings were taken to measure TSH, freeT4, freeT3, TRAb, CTX-I, 25OHD3, calcium, phosphate and PTH at baseline and at 6 weeks, 3, 6, 12 and 24 months after diagnosis and treatment start. We also measured 1.25OH2D3 at the first four visits. Vitamin D-binding protein (DBP) was measured at visits 1, 5 and 6, and sexual hormone binding globulin (SHBG) was measured at visits 1 and 6. During visit 1, the patients completed a questionnaire regarding risk factors for osteoporosis. Dual-energy X-ray absorptiometry (DXA) was performed on the hip and lumbar spine within 2 weeks after visit 1 and then 1 and 2 years after study inclusion. All 30 patients included in the study received conventional block and replace treatment. Anti-thyroid drugs, methimazole or propylthiouracil, were initiated in conjunction with visit 1, started with 10–20 mg methimazole doses or 150–300 mg propylthiouracil daily. Thyroxine 50–100 μg was added daily when thyroid hormone levels fell into the normal range, and TSH levels were kept in the low normal range. During follow-up, four patients received radioiodine (RAI) because of persistently elevated TRAb after 10–15 month treatment. One patient received RAI after 5 months at the request of the patient. Three patients underwent total thyroidectomy, two patients due to neutropenia, which developed directly after the start of methimazole in one case and after 10 months on methimazole in the other. One female patient underwent surgery after 5 months because of desire to become pregnant. Out of the remaining 22 subjects, 21 patients received ATD for 18–24 months until negative TRAb. One patient had a spontaneous recovery before treatment initiation.

Graves` orbitopathy (GO) was defined as signs and eye symptoms related to GD and classified as ‘mild’ GO with symptoms, such as gritty sensation and tearing due to dry eyes, caruncle swelling and/or redness, upper eyelid retraction, or as ‘moderate to severe’ in the instance of redness and/or swelling of the eyelids, pressure or pain in the eyes, exophthalmos, chemosis, diplopia or signs of optic nerve compression. The eye signs and symptoms were detailed in the medical records by the attending endocrinologist as well as by the nurse at all visits.

### Assays

Plasma TSH (reference interval 0.4–4.0 mIU/L), free T4 (reference interval 12–22 pmol/L), free T3 (reference interval 3.1–6.8 pmol/L), TRAb (reference < 1.75 IE/L), and CrossLaps^®^ (CTX-I) was analyzed using Cobas® pro instruments and reagents (Roche Diagnostics, Rotkreuz, Switzerland). An ELISA was used for the quantification of C-terminal telopeptides degradation products of Type I collagen (reference pre-menopause < 580 ng/L, post-menopause < 1000 ng/L), according to manufacturer (BioVendor). Plasma calcium (reference 2.15–2.5 mmol/L); serum PTH (reference interval 1.6–6.9 pmol/L); serum 25OHD3 (deficiency < 25 nmol/L and insufficiency < 50 nmol/L); serum 1,25OH2D3 (reference interval 60–210 pmol/L); plasma phosphate (reference interval 0.8–1.5 mmol/L); SHBG levels (reference interval for women 16–50 years: 26–110 nmol/L, > 50 years: 14–70 nmol/L and for men 14–48 nmol/L) were all measured per routine methods used at the Department of Clinical Chemistry at the Uppsala University Hospital. Vitamin D binding protein was measured by colorimetric sandwich-ELISA (Quantikine ELISA Human Vitamin D BP Immunoassay, catalogue number DVDBP0B; Lot P252667, R&D Systems).

### Statistical analysis

Data are presented as median (range) unless otherwise indicated. Changes in plasma levels of bone turnover markers and bone mineral density (BMD) were analysed using repeated-measures ANOVA with Greenhouse–Geisser correction. We performed post-hoc paired *t* tests compared with visit 1 using Bonferroni-adjusted alpha levels. For each analysis, normal distribution was assessed by Shapiro–Wilkes test at each timepoint. If the data were not normally distributed, they were log transformed (base 10), after which the assessment was reiterated. No extreme outliers were found after visual inspection of the data and the residuals. Due to violation of normality even after log transformation, changes in free T3 and DBP were analysed using Friedman’s test with post-hoc Bonferroni-adjusted Wilcoxon signed rank tests. For the same reason, changes in SHBG levels were analysed using Wilcoxon signed rank test. *P* values < the Bonferroni-adjusted alpha level (0.05/*n*, where n is number of hypotheses tested) were considered significant. SPSS version 27 (IBM Corp, Armonk, NY, USA) was used for the analyses.

## Results

Thirty subjects with overt hyperthyroidism without vitamin D supplementation or treatment against osteoporosis at diagnosis of GD were included in the study. Median age was 55 years (range 35–72 years), all but one was of Scandinavian origin, two were smokers, 29 were women. The baseline data present all 30 included patients (Table 1). Twenty-seven patients were included in the follow-up analyses after exclusion of those who started with vitamin D supplementation after baseline. Menopause was estimated as being at age 52 or above; no FSH data were available.

**Table 1:** Patient characteristics and concomitant medications at diagnosis

	*N*
Gender	29 females, 1 male
Age	55.5 (30–89) median and range
Menopause (> 52 years)	19
Smoking	2
Steroids	0
Estrogen	0
Biphosphonates	0
Vitamin D supplementation	0

### 25OHD3 and 1,25OH2D3 at baseline and during treatment

**Fig. 1:**
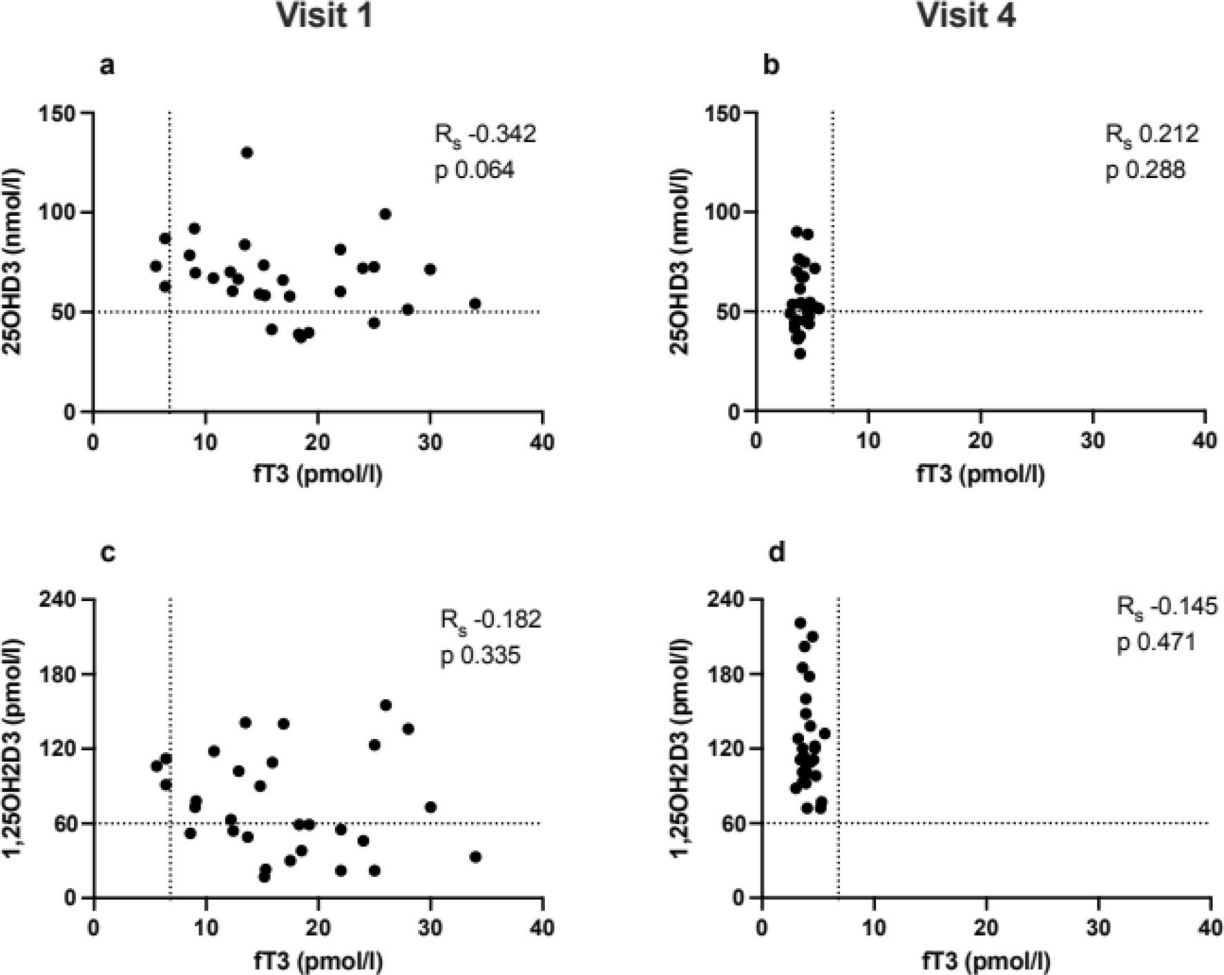
25OHD3 and 1,25OH2D3 vs free-T3 at visit 1 (*n* = 30) (**a**, **c**) and 4 (*n* = 27) (**b**, **d**). Vertical dashed line represents 6.8 pmol/l, the upper reference limit for free-T3 with the laboratory method that was utilised. *R*_*s*_ = Spearman Coefficient. Median and range for 25OHD3 were 66.8 (37.3–130) and 52.8 (28.8–90.1) at visit 1 and visit 4, respectively. Median and range for 1,25OH2D3 were 68 (17–155) and 119.5 (72–221) at visit 1 and visit 4, respectively

**Fig. 2:**
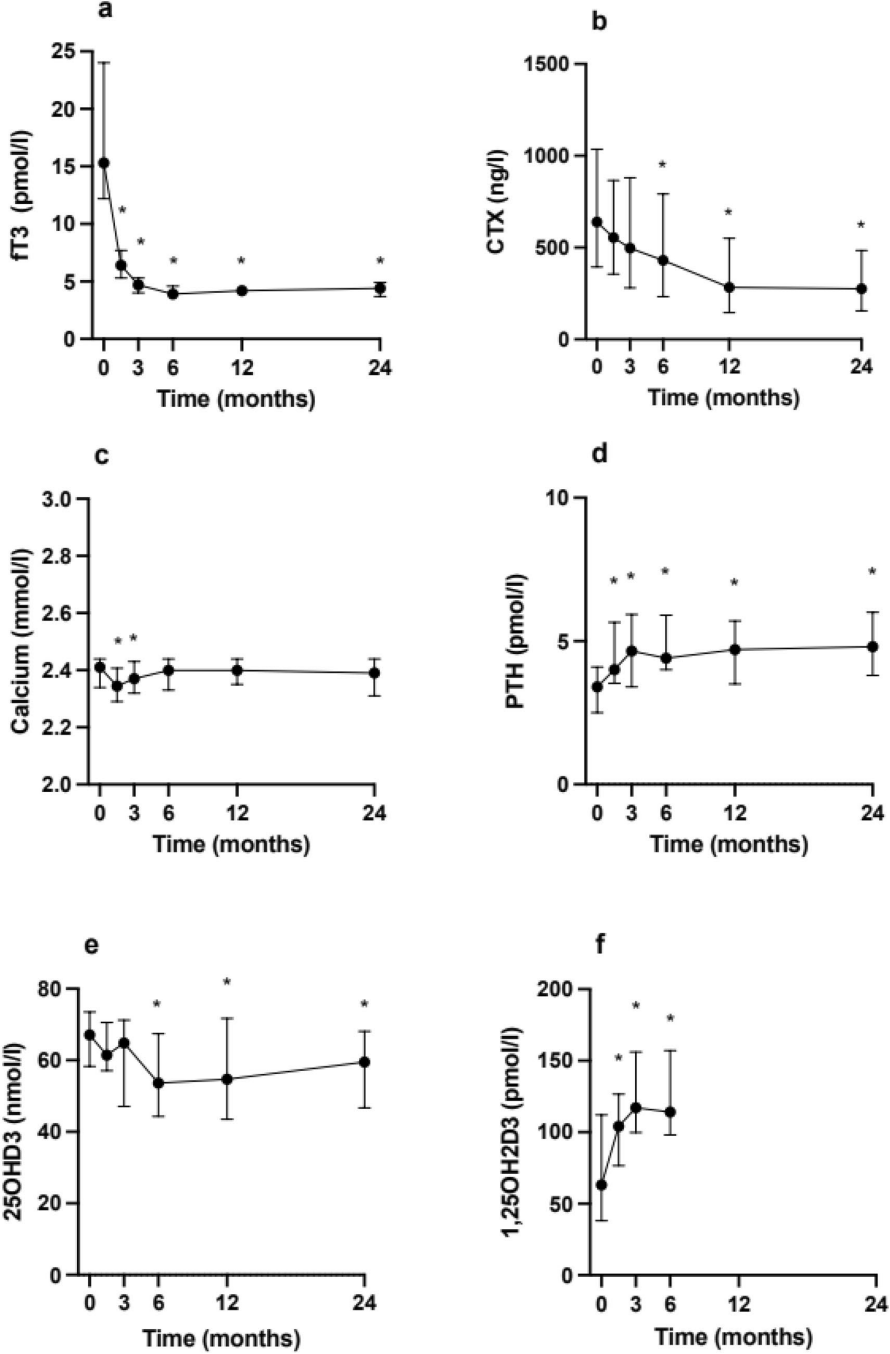
Levels of free-T3 (**a**) CTX (**b**), calcium (**c**), PTH (**d**), 25(OH)D3 (**e**), 1,25(OH)D3 (**f**) during the follow-up period of 24 months. Data are presented as median and interquartile range. Repeated measures analyses generated *p* < 0.01 for **b**–**f** (RMANOVA) and *p* < 0.001 for a (Friedman’s test). *p < Bonferroni-adjusted a-level (0.010 for all panels, except d where 0.013) for post-hoc Wilcoxon signed rank test (**a**) or paired *t* test (**b**–**f**) compared to visit 1 (at 0 months). CTX and PTH have been log transformed prior to analysis. Missing data points: 1/162 (**a**, **b**), 2/162 (**c**, **e**), 3/162 (**d**), 15/108 (**f**). Complete cases were 26/27 (**a**, b) 25/27 (**c**, **e**), 24/27 (**d**) and 17/27 (**f**, all had samples at baseline and visit 4). Only complete cases were included in the repeated measures analyses

At baseline, levels of 25OHD3 were 66.8 (37.3–130) nmol/L (Table 2); moreover, four patients had values below 50 nmol/L (Fig. 1a). There were no correlations between 25OHD3 and free T3 values (Fig. 1a), nor to TSH, TRAb or free T4 (data not shown). During treatment, values of free T3 became normalised, and the levels of 25OHD3 declined significantly (Figs. 1b, 2e, Table 2). At baseline, levels of 1,25OH2D3 were 68 (17–155) pmol/L (Table 2); moreover, 14 patients had 1,25OH2D3 levels below the lower reference range (Fig. 1c). During treatment, there was a significant increase in the levels of 1,25OH2D3 (Figs. 1d, 2f, Table 2). There was a negative correlation between 1,25OH2D3 and free T4 at visit 4 (Rs − 0.394, *p* = 0.042). Otherwise, there were no correlations between 1,25OH2D3 and free T3, TSH, TRAb or free T4 (data not shown).

**Table 2:** Thyroid hormone, Trab, Crosslaps, calcium, PTH, 25OHD3, 1,25OH2D3 and phosphate, levels (median and range) at diagnosis of and follow-up during 24 months of 30 patients with de novo GD

	Baseline	6 w	3 m	6 m	12 m	24 m
S-TSH	0.005 (0.005–0.04)	0.005 (0.005–0.911)	0.01 (0.005–5.68)	0.76 (0.005–10.4)	0.91 (0.005–4.67)	1.26 (0.07–4.47)
S-fT4	41 (17.6–100)	23 (13.9–49)	19.4 (11.2–33)	17.4 (11–31)	18.4 (12.8–30)	16.7 (10–27)
S-fT3	15.6 (5.6–34)	6.2 (3–12.1)	4.6 (3.4–12.6)	3.9 (3–5.6)	4.2 (3.3–6.1)	4.5 (3–6.9)
S-TRAb	5 (1.7–78)	5.8 (1.2–83)	3.8 (0.3–60)	1.5 (0.3–39)	0.4 (0.3–38)	0.3 (0.3–8.3)
S-Crosslaps	644.5 (161–1829)	542 (253–1566)	508 (147–2052)	477 (141–1748)	302 (87.5–970)	280 (97.1–895)
P-calcium	2.4 (2.3–2.6)	2.34 (2.1–2.5)	2.38 (2.2–2.5)	2.4 (2.1–2.5)	2.4 (2.2–2.6)	2.39 (2.2–2.6)
P-PTH	3.4 (2–6.1)	4 (2.5–9.7)	4.85 (2.4–8.1)	4.4 (2.2–10)	4.9 (2.4–9.2)	4.8 (2.4–10.6)
S-25OHD3	66.8 (37.3–130)	60.8 (30.2–108)	60.3 (27.4–102)	52 (28.8–90.1)	52.2 (28.2–93.8)	59.4 (29.1–88.8)
S-1,25OH2D3	68 (17–155)	104 (22–224)	117 (57–236)	114 (72–221)		
P-Phosphate	1.1 (0.79–1.63)	1.0 (0.61–1.36)	1.1 (0.77–1.36)	1.1 (0.75–1.46)	1.1 (0.77–1.33)	1.0 (0.73–1.33)

### Vitamin D binding protein and SHBG

During treatment, the levels of DBP did not change (*p* = 0.77), whereas SHBG declined from 172 (65–634) at baseline to 69.5 (14–299) at visit 6 (*p* < 0.001) (data not shown).

### Measures of calcium homeostasis

During the treatment, the levels of 25OHD3 significantly declined, whereas the levels of 1,25OH2D3 increased (Fig. 2e, f, Table 2). There was an initial and transient decrease in s-Ca and an increase in PTH (Fig. 2d, Table 2), and there was also a decrease in s-phosphate from 1.13 (0.79–1.63) at baseline to 1.0 (0.73–1.33) at visit 6 (Table 2). Regarding marker of bone resorption, there was a marked decrease in CTX-I (Fig. 2b, Table 2).

When calculating calcium values regarding fT4 at baseline using Spearman’s Rho the value of *r*_s_ = 0.20257, *p* (2-tailed) = 0.28. Although a positive trend but the relationship between the variables is not significant.

### Bone mineral density

**Fig. 3:**
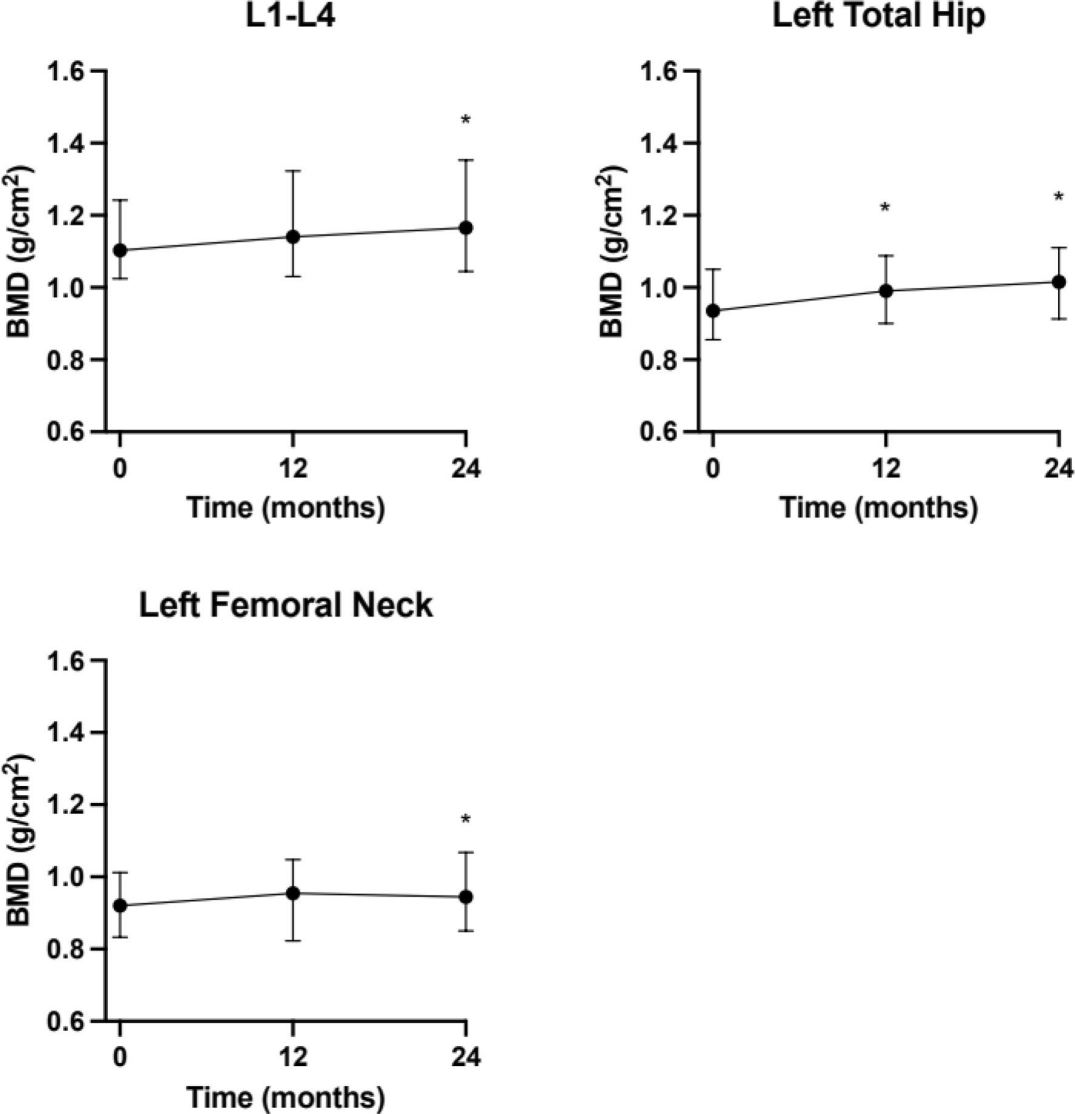
Bone mineral density measured by DXA during the follow-up period of 24 months. Data are presented as median and interquartile range. RMANOVA generated *p* = 0.008 for L1–L4, *p* < 0.001 for the left total hip and *p* = 0.010 for the left femoral neck

Bone mineral density was analysed by DXA at baseline and after 12 and 24 months, respectively. At baseline, the mean levels of BMD were 1.12 (± 0.17) g/cm^2^ in the spine, 0.95 (± 0.14) g/cm^2^ in the left total hip and 0.91 (± 0.13) g/cm^2^ in the left femoral neck (Fig. 3). The mean values regarding z scores were: 0.13 (± 1.3), 0.05 (± 1.07) and 0.006 (± 0.7) and T-score − 0.48 (± 1.4), − 0.5 (± 1.17) and − 0.5 (± 1.17), respectively (data not shown). There was only one patient with a T-score lower than − 2.5 in the spine.

Two years after treatment, the mean BMD increased significantly at all locations, in the spine 5.3% (± 7.5) (*p* = 0.002), in the left total hip 7.5% (± 7.9) (*p* < 0.001) and in the left femoral neck 4.4% (± 7.0) (*p* = 0.005) (Fig. 3). Explorative exclusion of the only male subject did not alter these results.

### Vitamin D and risk for GO

During the study 17 patients developed GO. Three patients with moderate to severe orbitopathy received steroids, two were treated with oral steroids and one with iv steroids. At baseline, there was no significant difference regarding the levels of 25OHD3 during the trial and risk of GO, 66.1 (37.3–99.2) and 67.1 (41.3–130) for GO and non-GO group, respectively (*p* = 0.5). Nor was there any difference of 1,25OH2D3, 59 (17–155) and 102 (22–136), respectively (*p* = 0.17).

## Discussion

In this prospective study on vitamin D, bone turnover and BMD in GD, there was no vitamin D deficiency at diagnosis. Furthermore, we could not detect any differences regarding the levels of 25OHD3 or 1.25OH2D3 at baseline and risk of developing GO. These data indicate that vitamin D deficiency is not associated with risk for developing GD or subsequent eye complications. GD had a significant impact on bone turnover and BMD, which was reversible after treatment of thyrotoxicosis. The data concerning the biochemical marker of bone metabolism CTX-I suggest that there should be a marked effect on bone density. This was demonstrated in this study.

Many studies have reported a correlation between vitamin D deficiency and different autoimmune diseases, including thyroid autoimmune diseases [31]. Vitamin D supplementation did not markedly reduce the recurrence of Graves’ disease, according to a newly published study [32]. In our study, 25OHD3 was significantly lower at follow-up than before treatment start with ATD. The data indicate that vitamin D deficiency is not prevalent in GD, nor are there any correlations between levels of vitamin D and levels of thyroid hormones. Regarding the correlation between free T4 and calcium at baseline, although a positive trend, the relationship between the variables was not significant. We believe that this might be related to the limited sample size. We found a negative correlation with free T4 at visit 4. However, this correlation was not very strong and especially when considering the multiple tests that were performed without formal correction could very well represent chance findings. We observed, however, a hitherto unshown shift in vitamin D metabolism during treatment, in that the levels of 25OHD3 decreased and the levels of 1,25OH2D3 increased. The levels of DBP did not differ from the levels at baseline, suggesting that the observed shift in 25OHD3 levels was unrelated to levels of vitamin D binding protein. Taken together, these data suggest that the shift in vitamin D metabolism was not related to GD but to the treatment, causing a decline in thyroid hormones. The reduction in thyroid hormones, thereby decreasing bone resorption and causing an increase in PTH, which, in turn, stimulates the conversion of 25OHD3 into its active form 1,25OH2D3, explains the lower levels of 25OHD3 after start of treatment. To be considered the half-life of 25OHD3 is 15 days [33], while the half-life of circulating 1,25OH2D is only 4–6 h [34].

As expected, BMD gradually increased after starting treatment, most prominent in the spine and total hip. These data are in line with the decreased CTX-I levels and suggest that the influence of thyrotoxicosis on bone density might be of a larger magnitude than previously believed, especially since the significant increase in BMD occurs despite an increase in PTH. The data on BMD in this study are congruent with a study from Australia, in which 15 female patients with active GD underwent BMD analysis by Dual-energy X-ray absorptiometry (DXA) at baseline and after 12 months with ATD. The bone mineral density increased significantly in the lumbar spine and femoral neck [35]. Two more studies [36, 37] have shown similar results.

In this study, we observe an increase in BMD concomitant with declining levels of 25OHD3. There are, however, results from randomised clinical studies, finding that calcium and vitamin D can be used in the treatment of osteoporosis [38]. Furthermore, patients with GD and low levels of 25OHD3 had the greatest improvement in the bone micro-architecture following treatment of hyperthyroidism [39]. Thus, the exact role of 25OHD3 as a predictor or therapeutic agent in osteoporosis, secondary to GD, needs further investigation. Data from this study imply that the increased conversion of 25OHD3 to 1,25OH2D3 might be a more relevant factor than the actual serum levels of 25OHD3.

Regarding the levels of vitamin D, the inclusion of patients was from February through November and blood test collected six times during the follow-up period for 2 years. The vitamin D levels in this study were similar to those measured in a cohort of 870 healthy Uppsala residents (52% females) [40] and higher than those reported in a study of 102 patients in the primary care [41]. We, therefore, believe that our cohort in this study does not deviate from the rest of the population regarding vitamin D levels.

The limitation of this study is the relatively small sample size. The strength, however, is the prospective design and the fact that not only 25OHD3 was analysed but also other parts of the calcium homeostasis. This includes the active form of vitamin D, 1,25OH2D3, which hitherto is less investigated in GD. Considering this, and that the reported findings reach statistical significance, we believe that the study offers sufficient strength for the conclusions presented.

In summary, no vitamin D deficiency was observed at time of diagnosis of Graves’ disease, and levels of 25OHD3 were significantly decreased at follow-up. Lower levels of 25OHD3 are likely to occur secondary to a higher conversion to 1,25(OH)D3 due to increased PTH levels after treatment of thyrotoxicosis.GD has a significant impact on the skeleton, and treatment of thyrotoxicosis leads to improved BMD, which continues to rise for 2 years after treatment start.
